# Biomechanical insights into carbon plate geometry in running shoes in male runners: implications for sprint performance and ankle stability

**DOI:** 10.3389/fbioe.2025.1735951

**Published:** 2026-01-07

**Authors:** Jin Teng, Meixi Pan, Yingfeng Han, Jorge Diaz-Cidoncha Garcia, Qing Yi, Siqin Shen

**Affiliations:** 1 Department of Sports Biomechanics, Beijing Sport University, Beijing, China; 2 College of Physical Education, Dalian University, Dalian, China; 3 Fédération Internationale de Football Association, Zurich, Switzerland

**Keywords:** ankle stability, biomechanics, carbon plate geometry, running shoes, sprints

## Abstract

To date, limited evidence exists on how carbon plate geometry influences sprint biomechanics. This study investigated the biomechanical effects of different carbon plate configurations in running shoes on sprint performance and lower-limb stability. Forty trained male sprinters performed submaximal sprinting at a controlled speed of 7 m s^-1^ while wearing shoes equipped with either a full-length carbon plate (FC) or a Y-shaped carbon plate (YC). Kinematic and kinetic data were collected using a motion capture system and force platform. Outcome measures included spatiotemporal variables, joint kinematics (hip, knee, ankle and metatarsophalangeal joint angles), and kinetics (vertical and horizontal ground reaction force and joint moment). Differences between the two shoe conditions were examined using paired-sample t-tests, while Statistical Parametric Mapping was applied to detect time-dependent differences across the stance phase. YC shoes demonstrated a higher peak vertical impact force, increasing from 2.90 to 3.14 BW (mean difference: 0.24 BW; Cohen’s d = 0.49; p = 0.003). Statistical Parametric Mapping further revealed sustained force elevations during 38%–65% of the stance phase (*p* < 0.001). In contrast, FC shoes demonstrated greater ankle eversion and reduced metatarsophalangeal extension (*p* < 0.001), suggesting FC shoes may improve energy efficiency but elevate eversion-related injury risk. In addition, YC shoes increased sagittal-plane ankle range of motion from 39.3° to 43.5° (mean difference: 4.2°; Cohen’s d = 0.75; *p* < 0.001), suggesting improved joint mobility demands during sprinting. These findings demonstrate that variations in carbon plate geometry lead to distinct alterations in lower-limb mechanical responses during sprinting in male runners, with different implications for force output, joint motion, and ankle control. This biomechanical evidence may assist in optimizing carbon plate design to balance sprint performance demands with ankle stability considerations.

## 1 Introduction

Sprinting represents a fundamental form of human locomotion and a critical determinant of athletic performance across multiple sports, including soccer, baseball, and rugby (Skoglund et al., 2023). The ability to accelerate rapidly and sustain high sprinting velocity is essential not only crucial for sprinters, but also a key component of successful performance in team sports that require repeated sprints (Bella et al., 2023). For example, short-distance sprints have been identified as the most frequent actions in scoring situations, executed by both goal scorers and supporting players (Faude et al., 2012). Additionally, team athletes with different competition standards exhibit varying sprinting abilities (Skoglund et al., 2023). Research also indicates that elite players become faster over time (Hisdal et al., 2013), suggesting that sprint performance enhancements require more attention and exploration.

As the main interface between the feet and the ground, running shoes was claimed to affect the biomechanical characteristics of the knee and ankle joints during running (TenBroek et al., 2014), which may further affect the risk of chronic sports injury (Sinclair, 2014). Due to technological advancements in structural and material engineering, running shoes with various functional characteristics are constantly being introduced, such as cushioning, stability, and minimalist running shoes (Davis, 2014; Sun et al., 2020).

There have been many studies on the biomechanical characteristics’ changes caused by the structure of running shoes. For instance, wearing shoes with heel cup protection for 4 weeks can decrease plantar pressure, plantar fascia stress, calcaneal stress, and self-perceived pain ratings (Li et al., 2018). Shoes with a greater heel-to-toe drop induce stronger knee flexion torque during the propulsion period and smaller knee extension torque during the cushion period (Besson et al., 2017), although different drops do not result in different injury risks (Malisoux et al., 2016). Soft midsole designs can reduce ground impact force and loading rate, thereby lowering the risk of impact-related injuries (Sterzing et al., 2013). Studies on shoelace design showed that a six-eyelet lacing pattern results in higher peak heel pressure and loading rate compared to a seven-eyelet lacing pattern, without differences in perceived comfort between the two patterns (Hagen et al., 2010).

In addition to the aforementioned, nowadays running shoe designs incorporate carbon fiber materials to increase running performance. Previous research revealed that carbon plates inserted at midsole could increase the bending stiffness, which could reduce metatarsophalangeal joint energy loss, thereby improving running performance and economy (Stefanyshyn and Nigg, 2000; Madden et al., 2016). Regarding the design of carbon plates, studies have compared and analysed the biomechanical effects of different carbon plate thicknesses and placement positions, which found that using thicker carbon plates (2 or 3 mm) close to the outsole can better alleviate plantar pressure (Song et al., 2023). More studies have reported on the advantages of carbon plates running shoes compared to regular running shoes, such as the Vaporfly 4% running shoes with inserted carbon plates, which could save 3.83%, 2.82%, and 2.70% of energy requirements on flat/uphill and downhill roads compared to traditional marathon running shoes with relatively thinner midsole (Whiting et al., 2022). However, there are no relevant reports on the differences in carbon plate shapes and designs, and previous studies have focused on typical jogging speeds (from 3.3 km/h to 3.61 km/h). Further exploration is needed for the biomechanical differences in running shoes designed with different carbon plate geometries, especially at a fast enough sprint speed.

Previous analysis has explored the effects of differential designed running shoes on risk factors related to chronic running pathology, but these biomechanical parameters are usually explored through discrete point analysis. For biomechanical parameters analysed according to time normalization, statistical parameter mapping (SPM) is more effective for analysis, because this method can detect data differences throughout the entire time window, thereby reducing the occurrence rate of Class II errors (Pataky et al., 2013). This study employs the SPM method to analyze the differences in lower limb biomechanical characteristics when wearing full-scale carbon plate geometry (FC) and Y-shaped carbon plate geometry (YC) during sprinting. We hypothesize that YC shoes provide better ankle stability during sprints, while FC shoes induce stronger longitudinal bending at the metatarsophalangeal joint.

## 2 Materials and methods

### 2.1 Participants

A total of Forty 100-m male sprinters who have reached the level of national second level athletes from Track and Field College [mean (SD) age = 23.7 (3.1) years, height = 1.75 (0.07) m, body mass = 69.6 (8.92) kg] were recruited before the test. All participants were required to wear two types of running shoes to sprint at supervised specific speed, and each participant had no history of lower extremity injuries in the past 6 months. None of the participants had previously worn the shoe model used in the experiment. Ethical approval was obtained by the Sports Science Experiments of Beijing Sport University Ethics Committee (reference: 2023284H) and a written informed consent form was obtained from every participant.

### 2.2 Footwear

**FIGURE 1:**
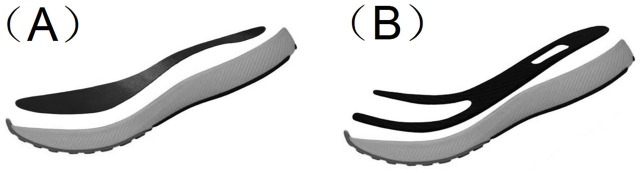
Structural illustration of the running shoes used in this study. **(A)** Full-length carbon plate design (FC). **(B)** Y-shaped carbon plate design (YC).

Two modified running shoes embedded with carbon plates in the midsole area were constructed for this study, as shown in Figure 1. Both carbon plates were fabricated with a uniform thickness of 2 mm and identical material properties, ensuring that the only variation between them was their shape. One of them was a full-length carbon plate design (FC) (Figure 1A), and the other was a Y-shaped carbon plate design (YC) (Figure 1B). The two shoe conditions were matched in terms of insole structure, material, outsole, and overall shoe weight to eliminate potential confounding factors. Both shoes were equipped with standard non-spiked outsoles featuring regular anti-slip patterns.

### 2.3 Movement tasks

**FIGURE 2:**
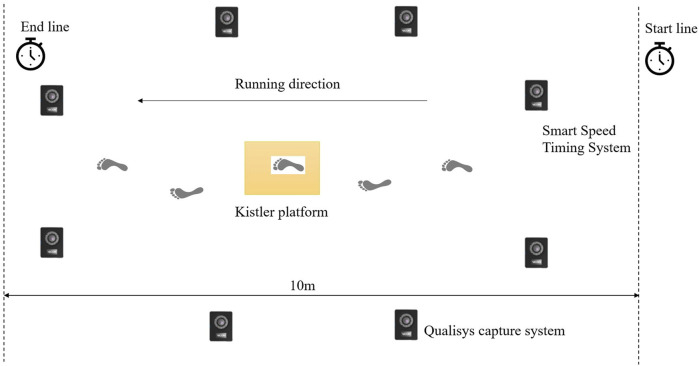
Schematic diagram of the experimental layout for sprint trials.

As shown in Figure 2, participants were instructed to sprint from an individual customed start position until passing through the collection area at a speed of 7 m/s with an error not exceeding 10%, which is s sprint speed that all participants can complete in the testing environment. Smart Speed timers were set at the starting and ending positions of the sprint, which are 10 m apart, to supervise the completion time (1.30–1.59s) and ensure consistent sprint speed (6.3–7.7 m/s). To ensure accurate data collection, the start position for each participant was calibrated so that the right foot landed on the force platform, which was integrated with the ground and surfaced with tartan. Unsuccessful trials, defined as missing the force platform, exceeding the specified time range, or displaying unnatural sprint movements, were repeated until five successful trials were collected for each shoe condition. The five valid strikes were subsequently averaged for analysis.

### 2.4 Procedures

Prior to the actual data acquisition, participants were instructed to perform 5 minutes of self-selected warm-up protocol and familiarize themselves with the testing protocol, especially the placement of the right foot on the force platform while sprinting. To measure the biomechanical parameters of the lower limbs, twenty-one reflective markers (9.5-mm diameter) were firmly affixed on the anatomical landmarks of the pelvis and right lower-limb as followed: left and right anterior superior iliac spines, left and right posterior superior iliac spines, lateral and medial epicondyles of the femur and malleolus, posterosuperior, posteroinferior and lateral of the calcaneus, the first and fifth metatarsal heads, and two panels inserted by four markers at the thigh and shank (Teng et al., 2022). Participants were instructed to tighten the shoelace to the last eyelet to ensure the tightness of the shoe was consistent (Lam et al., 2019).

For the actual tests, participants were asked to perform four trials for presentable maximum sprint, in both test shoe conditions (YC/FC). A Kistler force platform (Kistler Instruments, Model 9281CA; Winterthur, Switzerland, 1000 Hz) was embedded at the center of the test area to collect ground reaction force data at 1000 Hz. Qualisys motion analysis system (Oqus 500, Qualisys AB, Gothenburg, Sweden, 200 Hz) consisted of nine cameras was used to measure the marker trajectories at 200 Hz. One-minute and 10-min breaks were prescribed between trials and between different shoe conditions to minimize the influence of fatigue (Lam et al., 2018). The sequence of wearing shoes was randomized across participants.

### 2.5 Data analysis

Kinematic and kinetic data are synchronously processed through Qualisys Track Manager software. Three frames of data before and after the missing data point were used to interpolate the missing markers (Sigward and Powers, 2006). The kinematic data was smoothed using a fourth-order Butterworth bidirectional filter with a cut-off frequency of 12 Hz, and the kinetic data were normalized to body mass (Nigg et al., 2009). After manually identified all marker trajectories using Qualisys Track Manager software, the data was transmitted to Visual3D software (C-Motion Inc, Ontario, Canada) to establish a human model. All kinematic data of the hip, knee, ankle, and metatarsophalangeal joints are quantified using Euler angles. The segmental coordinate systems of the thigh and shank were determined based on the standing trial, during which participants were instructed to stand up straight with their legs shoulder-width apart, and all segments were arranged according to the coordinate system (Nigg et al., 2009).

The instance of touch down and toe-off were determined when the vGRF first exceeded 10 N (touch down) and below 10 N (toe-off) (Lam et al., 2017). The stance phase was subdivided based on the instant of maximum knee flexion (Wing et al., 2015). Specifically, the period from initial ground contact to the instant of maximum knee flexion was defined as the cushioning phase, whereas the period from maximum knee flexion to toe-off was defined as the propulsion phase. The maximum knee flexion event was applied in the analysis but was not explicitly marked in Figures for visual simplicity. Peak ground reaction forces were normalized to body weight (BW). Ankle and knee joint moments were calculated using inverse dynamics and subsequently normalized to body mass (Nm/kg) (Zhang et al., 2012).

### 2.6 Statistical analysis

Five repetitions were used in the analysis for kinematic and kinetic data. Differences in kinematic and kinetic parameters between the two shoe conditions were examined using paired-sample t-tests. The level of significance was set at α = 0.05, and effect sizes (Cohen’s d) were calculated to assess the magnitude of differences. All statistical analyses were conducted in SPSS (Version 26.0, IBM Corp., Armonk, NY, United States of America).

Following data processing, kinematics (hip, knee, ankle and metatarsophalangeal joints), and vertical ground reaction force during the entire stance phase were temporally normalized using linear interpolation to 101 data points. SPM was implemented in a hierarchical manner (Pataky et al., 2013), analogous to a one-way repeated-measures ANOVA with *post hoc* t-tests. The entire waveform was first examined using the omnibus SPM{F} test, and *post hoc* SPM{t} tests were subsequently performed when the omnibus effect reached significance. The default SPM thresholding approach was applied, which intrinsically controls the family-wise error rate based on random field theory. No additional correction (e.g., Bonferroni adjustment) was required because SPM’s cluster-level inference accounts for multiple comparisons across the continuum.

## 3 Result

As shown in Table 1, no significant differences were observed between YC and FC shoes in total foot contact time, cushion time, propulsion time, peak braking horizontal force, peak propulsion horizontal force, peak plantarflexion moment, or peak knee extension moment (all p > 0.05). However, the peak vertical impact force was significantly lower in the FC shoes compared with the YC shoes (2.90 ± 0.291 BW vs. 3.14 ± 0.447 BW, *p* = 0.003, d = 0.49).

**TABLE 1:** Spatiotemporal and kinetic variables (mean and standard deviation) in shoes with YC and FC.

Variables	YC	FC	Cohen’s d	*P* Value
Total foot contact time (ms)	136.5 (15.1)	138.7 (15.0)	0.20	0.214
Cushion time (ms)	60.2 (18.8)	65.2 (14.8)	0.22	0.131
Propulsion time (ms)	76.3 (19.2)	73.5 (13.3)	0.15	0.372
Peak braking horizontal force (BW)	0.731 (0.115)	0.726 (0.143)	0.04	0.815
Peak propulsion horizontal force (BW)	0.724 (0.104)	0.703 (0.220)	0.07	0.776
Peak impact vertical force (BW)	3.14 (0.447)	2.90 (0.291)	0.49	**0.003**
Peak plantarflexion moment (N/kg)	3.86 (1.20)	3.97 (1.47)	0.11	0.501
Peak knee extension moment (N/kg)	8.57 (3.49)	8.44 (4.03)	0.09	0.662

Bold values indicate statistically significant differences (P < 0.05).

During sprinting, significant differences in ankle joint kinematics were observed between the two shoe conditions. Compared with YC, FC shoes showed a significantly reduced plantarflexion angle at toe-off (20.7° ± 4.94° vs. 25.1° ± 5.56°, *p* < 0.001, d = 1.18) and peak plantarflexion angle (20.9° ± 4.92° vs. 25.4° ± 5.32°, *p* < 0.001, d = 1.08). Moreover, the ankle sagittal range of motion was significantly smaller in the FC condition (39.3° ± 5.65°) compared with the YC condition (43.5° ± 5.46°) (*p* < 0.001, d = 0.75). No significant differences were found in knee flexion angles at touchdown or toe-off, plantarflexion angle at touchdown, or peak dorsiflexion angle (all *p* > 0.05). The detailed results are presented in Table 2.

**TABLE 2:** Kinematic variables (mean and standard deviation) in shoes with YC and FC (°).

Variables	YC	FC	Cohen’s d	*P* Value
Knee flexion angle at touch down	34.0 (8.63)	34.1 (10.2)	0.01	0.938
Knee flexion angle at toe off	20.6 (6.77)	20.9 (6.92)	0.07	0.642
Plantarflexion angle at touch down	5.82 (10.8)	3.02 (10.3)	0.31	0.057
Plantarflexion angle at toe off	25.1 (5.56)	20.7 (4.94)	1.18	**0.000**
Peak dorsiflexion angle	17.9 (4.71)	18.4 (5.38)	0.09	0.559
Peak plantarflexion angle	25.4 (5.32)	20.9 (4.92)	1.08	**0.000**
Ankle sagittal range of motion	43.5 (5.46)	39.3 (5.65)	0.75	**0.000**
Peak knee extension angle	20.4 (6.47)	20.6 (6.48)	0.04	0.785
Peak knee flexion angle	47.7 (9.58)	48.2 (8.99)	0.08	0.608
Knee sagittal range of motion	27.3 (8.09)	27.6 (7.13)	0.06	0.724

Bold values indicate statistically significant differences (P < 0.05).

**FIGURE 3:**
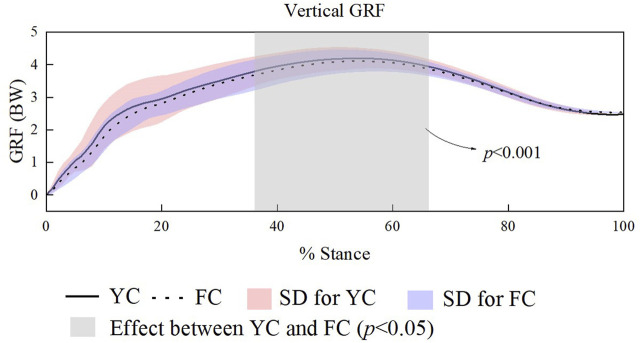
SPM-based comparison of vertical ground reaction force across the stance phase in male runners.

Further SPM analysis indicated a significant difference emerged in the vertical ground reaction force. As illustrated in Figure 3, this difference occurred between 38% and 65% of the stance phase, during which athletes wearing YC experienced significantly greater vertical ground reaction forces compared with those wearing FC (α = 0.05, t * = 3.296).

**FIGURE 4:**
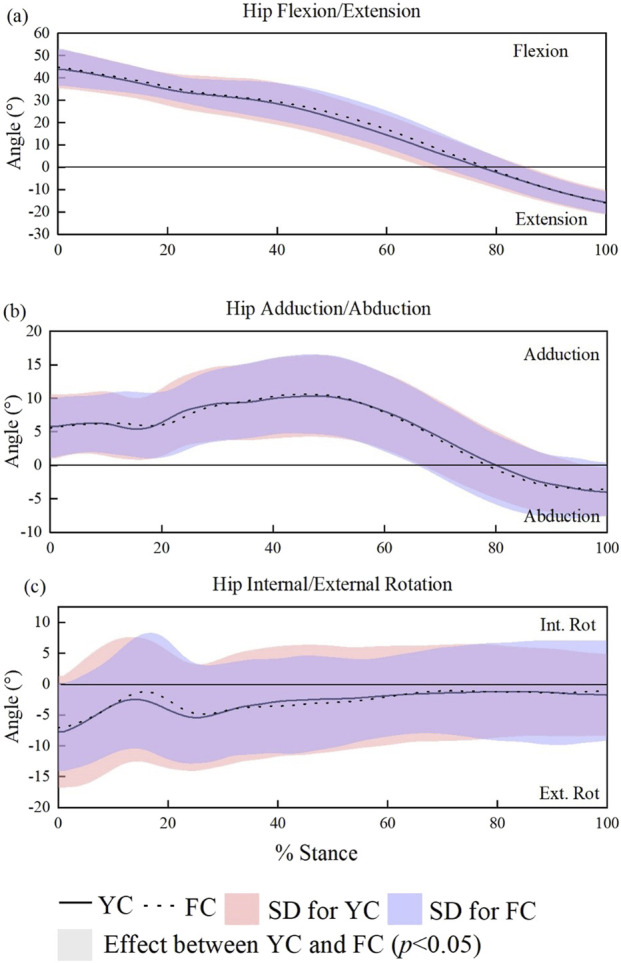
SPM-based comparison of sagittal, frontal, and transverse plane hip joint angles across the stance phase in male runners. **(a–c)**.

In terms of the kinematic parameters of the hip joint, Figure 4 shows the angle curve changes in the sagittal, frontal, and horizontal planes of the hip joint during a stance time. The data shows that there is no statistical difference in the kinematic parameters of the hip joint between sprinters wearing YC and FC during the entire stance time.

**FIGURE 5:**
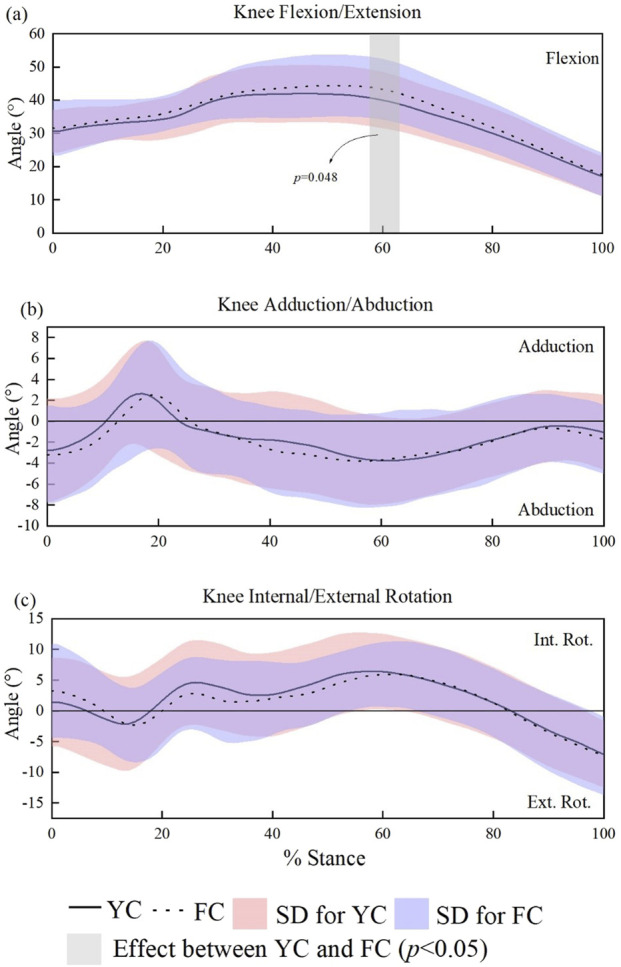
SPM-based comparison of sagittal, frontal, and transverse plane knee joint angles across the stance phase in male runners. **(a–c)**.

In terms of knee joint kinematics parameters as shown in Figure 5, only a short period (58%–63%) of a stance time shows different between two conditions. Specifically, wearing FC during sprinting induce a significantly larger knee flexion angle compared to YC (α = 0.05, t * = 2.949). However, there was no significant difference between the two pairs of shoes in terms of knee joint adduction/abduction and internal/external rotation.

**FIGURE 6:**
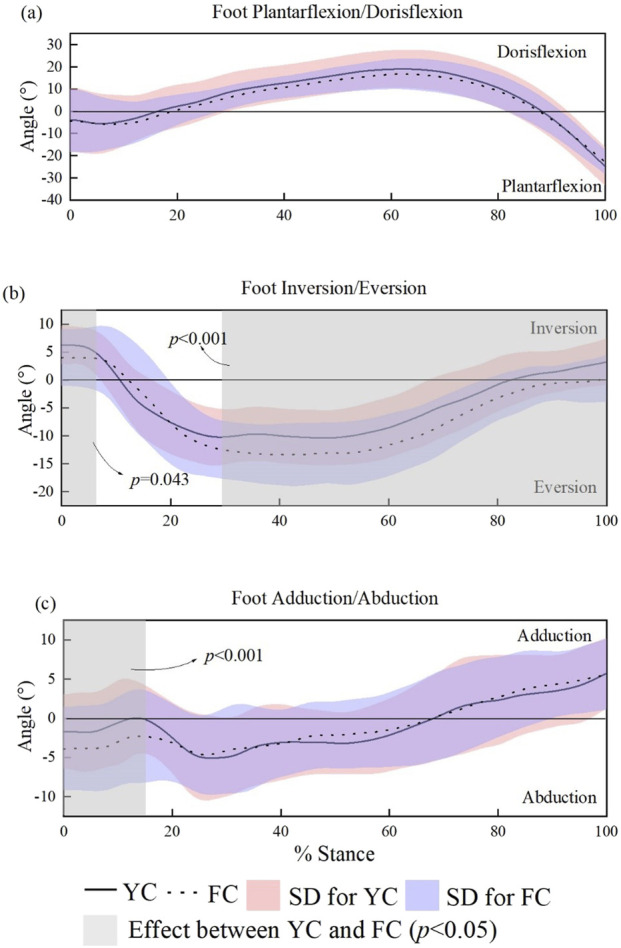
SPM-based comparison of sagittal, frontal, and transverse plane ankle joint angles across the stance phase in male runners. **(a–c)**.

The shape of carbon plate induced significantly impact on ankle joint kinematic variables, as shown in Figure 6. Specially, in the initial and the vast majority (30%–100%) period of stance time during sprinting, there is a significantly greater tendency of ankle eversion when wearing FC compared to the YC (α = 0.05, t * = 3.138), and wearing YC induces a more pronation trend in the 0%–15% stance period (α = 0.05, t * = 3.230); (Figure 6); But there was no significant difference between the two pairs of shoes regarding range of motion in plantar/dorsiflexion.

**FIGURE 7:**
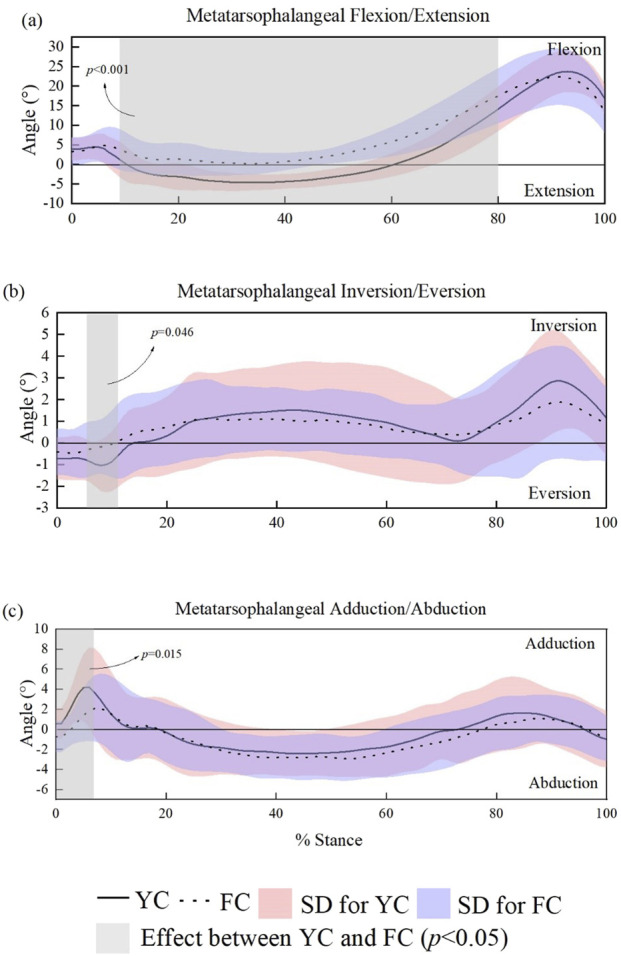
SPM-based comparison of sagittal, frontal, and transverse plane metatarsophalangeal joint angles across the stance phase in male runners. **(a–c)**.

With regard to metatarsophalangeal joint kinematic variables, the metatarsophalangeal joint results in a significantly greater extending trend when wearing YC than FC (α = 0.05, t * = 3.198) during the 10%–80% period of a stance time as shown in Figure 7, but no difference was detected between two shoe conditions regarding inversion/eversion and abduction/adduction curves, except only a short period (less than 10%) of stance time.

## 4 Discussion

In this study, no significant differences were observed between FC and YC shoes in most spatiotemporal and kinetic parameters, such as foot contact time, braking and propulsion horizontal forces, or knee extension moment. This indicates that the two plate geometries did not substantially alter the global timing or force generation patterns of sprinting. However, a notable difference was found in the vertical impact force, where FC induced significantly lower peak vertical impact forces compared with YC. This reduction is likely related to the greater longitudinal stiffness of the full-length carbon plate, which may distribute loads more evenly across the stance phase and thereby attenuate the magnitude of impact at initial contact. Previous research also revealed stiffer shoes induced less impact loading, rather than the maximalist shoes (Kulmala et al., 2018), supporting the present findings.

In terms of joint kinematics, FC resulted in a smaller peak plantarflexion angle and reduced sagittal-plane ankle range of motion compared with YC. These alterations suggest that the full-length plate constrains ankle mobility, which may improve energy transfer efficiency but potentially compromise natural joint motion and increase demands on surrounding stabilizing structures. Conversely, the YC configuration allowed for greater plantarflexion and range of motion, which may facilitate propulsion but could also increase mechanical demands on the plantar fascia and Achilles tendon (Sinclair et al., 2021).

Taken together, these results highlight distinct biomechanical responses associated with different plate geometries: the FC shoe appears to enhance impact attenuation and motion control, whereas the YC shoe favours greater ankle mobility and push-off potential. From a practical perspective, this implies that shoe design should be tailored to athlete profiles and training needs: FC-type shoes may better serve athletes prioritizing energy efficiency and impact reduction, whereas YC-type shoes may be advantageous for athletes seeking enhanced ankle flexibility and propulsion capacity (Esculier et al., 2015).

Furthermore, this study used the SPM method to compare the biomechanical characteristics between a full-scale carbon plate designed running shoes (FC) and a Y-shaped carbon plate designed running shoes (YC). The joint angle changes of the hip, knee, ankle, and metatarsophalangeal joints, as well as the vertical ground reaction force, were analysed and compared. The advantage of SPM is that it allows continuous comparison across the entire stance phase, rather than focusing only on discrete values, thereby providing a more detailed description of temporal differences.

The vertical ground reaction force curves indicated consistent general patterns between the two shoes. Nevertheless, SPM revealed significantly greater vertical GRF in YC than FC between 38% and 65% of the stance phase (*p* < 0.001). This may be attributed to the higher longitudinal stiffness of the full-scale carbon plate in FC shoes, which shifts the center of pressure forward during the push-off phase, thereby increasing the joint moment arm and decreasing the ground reaction forces (GRFs) (Oh and Park, 2017). Supporting this interpretation, previous studies also reported that vertical impact peaks were lower in stiff shoe conditions compared with softer shoes during treadmill (Malisoux et al., 2023) and the runway (Pollard et al., 2018).

Wearing the two types of running shoes has no statistical effect on hip and knee joint kinematics during stance, except for a small transient difference in knee extension. These results are consistent with prior studies, which generally suggest that carbon plates primarily affect metatarsophalangeal and ankle function, with limited influence on hip and knee mechanics (Willwacher et al., 2014; Madden et al., 2016; Oh and Park, 2017).

The ankle sagittal-plane angle curves showed no differences in the plantarflexion–dorsiflexion transition. However, in the frontal plane, FC induced significantly greater eversion amplitude than YC throughout 30%–100% of stance. In this study, ankle stability was operationally defined using the eversion angle during stance, with larger eversion amplitudes interpreted as indicative of reduced mechanical stability at the subtalar joint. Greater eversion excursion may impair ankle stability, potentially increasing the risk of inversion-related injuries (Hannigan and Pollard, 2020). This result also supports the original hypothesis that wearing YC shoes can ensure better ankle stability during sprints. The poor stability of FC shoes may be related to stiffer midsole by the full-scale carbon plate, even if a previous paper claimed the maximal ankle eversion angle was consistent between different stiffness shoes conditions (Malisoux et al., 2023). One plausible explanation is that the sprinting speed (25.2 km/h) adopted here is much faster than the speed in Malisoux’s research (10 km/h), and the higher intensity sprints might amplify the instability differences caused by the stiffness of the shoes.

The results of this study revealed greater metatarsophalangeal joint flexion and extension amplitude while wearing YC than FC shoes. Specifically, YC shoes induced a stronger extension tendency at the metatarsophalangeal joint during 10%–80% of stance, while FC shoes maintained a more flexed configuration throughout stance. Reduced metatarsophalangeal mobility at the metatarsophalangeal joint was related with a stronger forefoot bending stiffness (Madden et al., 2016), so the design of a full-scale carbon plate induced stronger forefoot bending stiffness compared to the Y-shaped carbon plate. Furthermore, previous research found that increasing the bending stiffness of the metatarsophalangeal joint could reduce energy loss at the joint, thereby achieving a positive impact on running performance (Stefanyshyn and Nigg, 2000). Accordingly, the FC shoe may promote energy efficiency by limiting forefoot motion, but this comes at the cost of restricted joint mobility and potentially increased local loading.

## 5 Conclusion and limitations

Overall, this study revealed that different shapes of carbon plates embedded in shoes resulted in different biomechanical characteristics during sprinting. Specifically, FC shoes with a full-scale carbon plate design exhibited higher longitudinal bending stiffness than YC shoes with a Y-shaped carbon plate, potentially reducing energy loss during sprinting. However, FC shoes induced greater frontal mobility of the ankle joint compared to YC shoes, which is relatively unfavourable for maintaining a stable neutral position of the ankle joint. Based on the above findings, running shoes with a full-scale carbon plate design could be a better choice for athletes who can adapt to the stiffer midsole, but sprinters with chronic ankle instability may prefer Y-shaped carbon plate designs or running shoes with relatively lower hardness.

This study employed a short-distance sprint protocol (∼10 m at 7 m/s), which may not fully represent typical competitive sprints or middle-to long-distance running scenarios. Therefore, caution should be taken when generalizing the findings beyond the tested condition. Because all participants were male sprinters, the findings may not generalize to female athletes, who often exhibit different ankle mechanics and joint laxity profiles, future research including both sexes is warranted to determine whether similar effects would be observed in female runners. Another limitation of this study is that the carbon fiber used here only differed in shape, as different production processes for carbon fiber materials can also significantly impact its mechanical properties. Future research can explore the differences brought by more types of carbon plates, curvatures, and other designs.

## Data Availability

The raw data supporting the conclusions of this article will be made available by the authors, without undue reservation.

## References

[B1] Bella L.D. Doma K. Sinclair W. H. Connor J. D. (2023). The acute effect of various feedback approaches on sprint performance, motivation, and affective mood states in highly trained female athletes: a randomized crossover trial. Int. J. Sports Physiol. Perform. 18, 313–319. 10.1123/ijspp.2022-0320 36750119

[B2] Besson T. Morio C. Rossi J. (2017). Effects of shoe drop on running mechanics in women. Comput. Methods Biomech. Biomed. Engin 20, 19–20. 10.1080/10255842.2017.1382840 29088632

[B3] Davis I. S. (2014). The re-emergence of the minimal running shoe. J. Orthop. Sports Phys. Ther. 44, 775–784. 10.2519/jospt.2014.5521 25211531

[B4] Esculier J. F. Dubois B. Dionne C. E. Leblond J. Roy J. S. (2015). A consensus definition and rating scale for minimalist shoes. J. Foot Ankle Res. 8, 1–9. 10.1186/s13047-015-0094-5 26300981 PMC4543477

[B5] Faude O. Koch T. Meyer T. (2012). Straight sprinting is the most frequent action in goal situations in professional football. J. Sports Sci. 30, 625–631. 10.1080/02640414.2012.665940 22394328

[B6] Hagen M. Homme A. K. Umlauf T. Hennig E. M. (2010). Effects of different shoe-lacing patterns on dorsal pressure distribution during running and perceived comfort. Res. Sports Med. 18, 176–187. 10.1080/15438627.2010.490180 20623434

[B7] Hannigan J. J. Pollard C. D. (2020). Differences in running biomechanics between a maximal, traditional, and minimal running shoe. J. Sci. Med. Sport 23, 15–19. 10.1016/j.jsams.2019.08.008 31501022

[B8] Hisdal J. Seiler S. Federation N. O. Sciences S. (2013). The role and development of sprinting speed in soccer. Int. J. Sports Physiol. Perform., 432–441. 10.1123/IJSPP.2013-0121 23982902

[B9] Kulmala J. P. Kosonen J. Nurminen J. Avela J. (2018). Running in highly cushioned shoes increases leg stiffness and amplifies impact loading. Sci. Rep. 8, 1–7. 10.1038/s41598-018-35980-6 30504822 PMC6269547

[B10] Lam W. K. Qu Y. Yang F. Cheung R. T. H. (2017). Do rotational shear-cushioning shoes influence horizontal ground reaction forces and perceived comfort during basketball cutting maneuvers? PeerJ 2017, 1–13. 10.7717/peerj.4086 29181281 PMC5702506

[B11] Lam W. K. Lee W. C. C. Lee W. M. Ma C. Z. H. Kong P. W. (2018). Segmented forefoot plate in basketball footwear: does it influence performance and foot joint kinematics and kinetics? J. Appl. Biomech. 34, 31–38. 10.1123/jab.2017-0044 28836881

[B12] Lam W. K. Kan W. H. Chia J. S. Kong P. W. (2019). Effect of shoe modifications on biomechanical changes in basketball: a systematic review. Sports Biomech. 00, 1–27. 10.1080/14763141.2019.1656770 31578122

[B13] Li L. Yang L. Yu F. Shi J. Zhu L. Yang X. (2018). 3D printing individualized heel cup for improving the self-reported pain of plantar fasciitis. J. Transl. Med. 16, 1–11. 10.1186/s12967-018-1547-y 29914501 PMC6007068

[B14] Madden R. Sakaguchi M. Tomaras E. K. Wannop J. W. Stefanyshyn D. (2016). Forefoot bending stiffness, running economy and kinematics during overground running. Footwear Sci. 8, 91–98. 10.1080/19424280.2015.1130754

[B15] Malisoux L. Chambon N. Urhausen A. Theisen D. (2016). Influence of the heel-to-toe drop of standard cushioned running shoes on injury risk in leisure-time runners. Am. J. Sports Med. 44, 2933–2940. 10.1177/0363546516654690 27501833

[B16] Malisoux L. Gette P. Backes A. Delattre N. Theisen D. (2023). Lower impact forces but greater burden for the musculoskeletal system in running shoes with greater cushioning stiffness. Eur. J. Sport Sci. 23, 210–220. 10.1080/17461391.2021.2023655 35014593

[B17] Nigg B. M. Stefanyshyn D. J. Rozitis A. I. Mündermann A. (2009). Resultant knee joint moments for lateral movement tasks on sliding and non-sliding sport surfaces. J. Sports Sci. 27, 427–435. 10.1080/02640410802669161 19253080

[B18] Oh K. Park S. (2017). The bending stiffness of shoes is beneficial to running energetics if it does not disturb the natural MTP joint flexion. J. Biomech. 53, 127–135. 10.1016/j.jbiomech.2017.01.014 28168959

[B19] Pataky T. C. Robinson M. A. Vanrenterghem J. (2013). Vector field statistical analysis of kinematic and force trajectories. J. Biomech. 46, 2394–2401. 10.1016/j.jbiomech.2013.07.031 23948374

[B20] Pollard C. D. Ter Har J. A. Hannigan J. J. Norcross M. F. (2018). Influence of maximal running shoes on biomechanics before and after a 5K run. Orthop. J. Sports Med. 6, 1–5. 10.1177/2325967118775720 29900183 PMC5992812

[B21] Sigward S. Powers C. M. (2006). The influence of experience on knee mechanics during side-step cutting in females. Clin. Biomech. 21, 740–747. 10.1016/j.clinbiomech.2006.03.003 16675083

[B22] Sinclair J. (2014). Effects of barefoot and barefoot inspired footwear on knee and ankle loading during running. Clin. Biomech. 29, 395–399. 10.1016/j.clinbiomech.2014.02.004 24636307

[B23] Sinclair J. Brooks D. Taylor P. J. Liles N. B. (2021). Effects of running in minimal, maximal and traditional running shoes: a musculoskeletal simulation exploration using statistical parametric mapping and Bayesian analyses. Footwear Sci. 13, 143–156. 10.1080/19424280.2021.1892834

[B24] Skoglund A. Strand M. F. Haugen T. A. (2023). The effect of flying sprints at 90% to 95% of maximal velocity on sprint performance. Int. J. Sports Physiol. Perform. 18, 248–254. 10.1123/ijspp.2022-0244 36649725

[B25] Song Y. Cen X. Chen H. Sun D. Munivrana G. Bálint K. (2023). The influence of running shoe with different carbon-fiber plate designs on internal foot mechanics: a pilot computational analysis. J. Biomech. 153, 111597. 10.1016/j.jbiomech.2023.111597 37126883

[B26] Stefanyshyn D. J. Nigg B. M. (2000). Influence of midsole bending stiffness on joint energy and jump height performance. Med. Sci. Sports Exerc 32, 471–476. 10.1097/00005768-200002000-00032 10694134

[B27] Sterzing T. Schweiger V. Ding R. Cheung J. T. M. Brauner T. (2013). Influence of rearfoot and forefoot midsole hardness on biomechanical and perception variables during heel-toe running. Footwear Sci. 5, 71–79. 10.1080/19424280.2012.757810

[B28] Sun X. Lam W. K. Zhang X. Wang J. Fu W. (2020). Systematic review of the role of footwear constructions in running biomechanics: implications for running-related injury and performance. J. Sports Sci. Med. 19, 20–37. 32132824 PMC7039038

[B29] TenBroek T. M. Rodrigues P. A. Frederick E. C. Hamill J. (2014). Midsole thickness affects running patterns in habitual rearfoot strikers during a sustained run. J. Appl. Biomech. 30, 521–528. 10.1123/jab.2012-0224 24615336

[B30] Teng J. Qu F. Shen S. Jia S. W. Lam W. K. (2022). Effects of midsole thickness on ground reaction force, ankle stability, and sports performances in four basketball movements. Sports Biomech. 00, 1–14. 10.1080/14763141.2022.2112747 36047733

[B31] Whiting C. S. Hoogkamer W. Kram R. (2022). Metabolic cost of level, uphill, and downhill running in highly cushioned shoes with carbon-fiber plates: graded running in modern marathon shoes. J. Sport Health Sci. 11, 303–308. 10.1016/j.jshs.2021.10.004 34740871 PMC9189710

[B32] Willwacher S. König M. Braunstein B. Goldmann J. P. Brüggemann G. P. (2014). The gearing function of running shoe longitudinal bending stiffness. Gait Posture 40, 386–390. 10.1016/j.gaitpost.2014.05.005 24882222

[B33] Wing G. Lam K. Park E. J. Lee K. Cheung J. T. Cheung M. A. N. (2015). Shoe collar height effect on athletic performance , ankle joint kinematics and kinetics during unanticipated maximum-effort side-cutting performance performance. J. Sports Sci. 33, 37–41. 10.1080/02640414.2015.1011206 25671398

[B34] Zhang S. Wortley M. Freedman J. Carson D. Paquette M. R. (2012). Do ankle braces provide similar effects on ankle biomechanical variables in subjects with and without chronic ankle instability during landing. JSHS 1, 114–120. 10.1016/j.jshs.2012.07.002

